# Exploring attentional bias towards threatening faces in chimpanzees using the dot probe task

**DOI:** 10.1371/journal.pone.0207378

**Published:** 2018-11-28

**Authors:** Duncan A. Wilson, Masaki Tomonaga

**Affiliations:** Primate Research Institute, Kyoto University, Inuyama, Aichi, Japan; University of California, San Francisco, UNITED STATES

## Abstract

Primates have evolved to rapidly detect and respond to danger in their environment. However, the mechanisms involved in attending to threatening stimuli are not fully understood. The dot-probe task is one of the most widely used experimental paradigms to investigate these mechanisms in humans. However, to date, few studies have been conducted in non-human primates. The aim of this study was to investigate whether the dot-probe task can measure attentional biases towards threatening faces in chimpanzees. Eight adult chimpanzees participated in a series of touch screen dot-probe tasks. We predicted faster response times towards chimpanzee threatening faces relative to neutral faces and faster response times towards faces of high threat intensity (scream) than low threat intensity (bared teeth). Contrary to prediction, response times for chimpanzee threatening faces relative to neutral faces did not differ. In addition, we found no difference in response times for faces of high and low threat intensity. In conclusion, we found no evidence that the touch screen dot-probe task can measure attentional biases specifically towards threatening faces in our chimpanzees. Methodological limitations of using the task to measure emotional attention in human and non-human primates, including stimulus threat intensity, emotional state, stimulus presentation duration and manual responding are discussed.

## Introduction

Faces are one of most important and salient social stimuli for primates. They convey information about identity, age, sex, attention and emotion [1]. Humans display a wide range of facial expressions to communicate their emotions, including anger, fear, disgust, sadness, surprise and happiness [2, 3]. Similarity in human and animal facial expressions have long been thought to reflect similarity in basic emotions [4, 5]. The animal fear system has evolved to rapidly detect and respond to danger in the environment and is particularly sensitive to threatening stimuli such as snakes and angry faces [6, 7]. Thus, information from threatening stimuli is given attentional priority over other information [8]. This attentional bias refers to “differential attentional allocation towards threatening stimuli relative to neutral stimuli” [9] (p. 204).

To investigate the mechanisms involved in attending to threatening stimuli in humans, several experimental paradigms have been developed. These include emotional stroop, visual search, attentional probe, spatial cueing and rapid serial visual presentation tasks [9, 10]. One of the most widely used spatial cueing tasks is the visual dot-probe task, originally developed by MacLeod et al. [11]. Two stimuli (words or images) are simultaneously presented on a screen for a short duration (traditionally 500 ms). Typically, one of the stimuli is threatening (angry face) and the other is neutral (non-expressive face). The stimuli disappear and a dot (probe) appears randomly in the spatial location of either the threatening stimulus (congruent trial) or the neutral stimulus (incongruent trial). Response times to detect the dot are recorded using computer keys. It is assumed if attention is already fixated in the spatial location of one stimulus, detecting the dot in the same location will result in faster response times [10]. Faster reaction times on congruent trials indicate attentional bias towards threatening faces (vigilance) and on incongruent trials attentional bias away from threatening faces (avoidance) [12].

The dot-probe task has been used extensively to investigate the relationship between emotion and attention in humans. Vigilance towards threatening faces is more consistently found in anxious than non-anxious people. However, whilst in some studies anxious participants show greater vigilance towards threatening faces compared with non-anxious participants [13–18], in other studies they show avoidance [19, 20] or no attentional bias [21–23]. Several methodological differences likely account for these inconsistencies. Two of the most important moderating variables for measuring vigilance towards threatening faces are the duration between the start of the stimulus and the start of the dot presentation (stimulus onset asynchrony; SOA) and stimulus threat intensity [9, 10, 24].

Several human studies have found vigilance towards threatening faces at short SOAs. Shorter SOAs are thought to facilitate threat detection by the amygdala and involve more automatic processing, whereas longer SOAs facilitate strategic processing by the prefrontal cortex and involve greater attentional control [9, 25]. Stevens et al. [18] found vigilance towards threatening faces at 175 ms but not 600 ms, Cooper and Langton [26] at 100 ms but not 500 ms, and Holmes et al. [27] at 30 ms or 100 ms but not 500 ms or 1,000 ms. Vigilance towards threatening faces has also been found at the longer SOA of 500 ms but not 1,250 ms [15, 28]. Overall, SOAs of less than 500 ms appear more suitable for measuring vigilance towards threatening faces. In addition, shorter SOAs prevent attention from switching between the two stimuli in the dot-probe task [25]. Eye saccades are “rapid, ballistic eye movements that move the fovea (the region of highest visual acuity) towards the target stimulus” [29] (p. 381) and can occur within 200 ms. Therefore, SOA duration should ideally be limited to 150 ms, to prevent attention from switching during stimuli presentation [29].

Regarding stimuli threat intensity, faces expressing anger are often used as stimuli, as they are more salient, threatening and ecologically valid than words [30, 31]. An interesting study by Wilson and MacLeod [32] investigated attentional bias to faces of different threat intensity in the dot-probe task. Low anxiety participants showed vigilance towards morphed faces expressing very high anger, but not moderate anger. High anxiety participants showed vigilance towards both very high and moderate anger faces. Overall, vigilance towards threatening faces increased with threat intensity. In a simplified dot-probe task in humans, de Valk et al. [33] found faster response times to touch angry faces than fearful or neutral faces. Although both angry and fearful faces signal danger in the environment, angry faces signal a more direct threat to the observer [34]. Therefore, angry faces likely have a higher threat intensity than fearful faces.

Despite extensive use of the dot-probe task to investigate emotional attention in humans, few studies have been conducted with animals [35]. Recently, a handful of dot-probe studies in non-human primates have been conducted using touchscreens. In monkeys, Koda et al. [36] presented two Japanese macaques with conspecific infant and adult faces for 100 ms. Attention was captured by visual cues, but no bias was found towards infant faces. In six rhesus monkeys King et al. [37] found vigilance towards threatening conspecific faces presented for 1,000 ms. Also in six rhesus monkeys Parr et al. [38] found vigilance towards conspecific threatening faces (bared-teeth and open mouth threat expressions) presented for 500 ms. In great apes, Tomonaga and Imura [39] conducted the first visuo-spatial cueing (dot-probe) experiment with three chimpanzees. Neutral chimpanzee and human faces and random objects were presented for 200 ms. Attentional biases were observed towards chimpanzee and human faces versus objects, but not bananas versus objects, indicating a face-specific bias. More recently, Kret et al. [40] presented four bonobos with images of conspecifics and other animals as control stimuli for 300 ms. Bonobo images consisted of either emotional scenes (i.e. distress, groom, sex, yawn, play, food, pant hoot) or neutral scenes, including the whole body. Attention was biased towards emotional scenes, with the strongest biases towards affiliative and protective behaviours such as sex, yawning and grooming. However, when Kret et al. [41] presented eight chimpanzees with conspecific whole-body threatening stimuli (fearful and display expressions) paired with neutral stimuli for 33 ms and 300 ms no vigilance towards threatening stimuli was found.

Overall, evidence from non-human primate studies suggests the dot-probe task can successfully measure attentional biases to emotional stimuli in monkeys and great apes. However, many methodological inconsistencies in both the human and non-human primate literature exist, including but not limited to: SOA duration, stimuli threat intensity, stimuli pair combination and response methods. Nevertheless, the task offers a promising method to investigate attentional processes in emotional perception in non-human primates. Furthermore, the task is implicit and requires little training, making it ideal for use with non-human primates and other animal species [24].

The aim of the present study was to investigate whether the dot-probe task can measure attentional biases towards threatening faces in chimpanzees. We predicted faster response times to touch the dot replacing threatening faces than neutral faces or scrambled faces. In addition, we predicted faster response times towards faces of higher than lower threat intensity [33, 34].

## Materials and methods

### Ethics statement

This study was approved by the Animal Welfare and Care Committee of the Primate Research Institute, Kyoto University, and the Animal Research Committee of Kyoto University (2016–064, 2017–106), and followed the *Guidelines for the Care and Use of Laboratory Primates* of the Primate Research Institute, Kyoto University (Version 3, 2010). No food or water deprivation was used in the study.

### Participants and housing

Eight adult chimpanzees (*Pan troglodytes*), six females and two males, participated in the study at the Primate Research Institute, Kyoto University, Japan (Table 1) [42]. The chimpanzees were members of a social group of 11 individuals living in an environmentally enriched facility consisting of two outdoor enclosures (250 m^2^ and 280 m^2^), an open air outdoor enclosure (700 m^2^) and indoor living rooms linked to testing rooms. The open air outdoor enclosure was equipped with 15 m high climbing frames and included trees [43, 44]. The chimpanzees had extensive experience participating in touchscreen cognitive tasks.

**Table 1:** Basic information about the eight chimpanzees.

Name	GAIN ID Number^a^	Sex	Age in years (at study start)
**Ai**	0434	Female	41
**Ayumu**	0608	Male	16
**Chloe**	0441	Female	37
**Cleo**	0609	Female	17
**Gon**	0437	Male	51
**Pal**	0611	Female	16
**Pendensa**	0095	Female	39
**Popo**	0438	Female	34

^a^ Identification number (ID) for each chimpanzee listed in the database of the Great Ape Information Network (GAIN): https://shigen.nig.ac.jp/gain/ [42].

### Apparatus

Experiments were conducted in an experimental booth (1.80 × 2.15 × 1.75 m) inside a testing room. Each chimpanzee voluntarily walked to the booth through an overhead walkway connected to the living rooms. Two 17-inch touch-sensitive LCD monitors (1280 × 1024 pixels) encased in Plexiglas were used to present visual stimuli at approximately 40 cm distance. Food rewards (8 mm apple cubes) were delivered via a universal feeder device. All experimental events were controlled by a PC and the computer task was programmed using Microsoft Visual Basic 2010 (Express Edition).

### Stimuli

Facial stimuli consisted of cropped photographs (200 × 250 pixels, 53 mm × 66 mm) of unfamiliar chimpanzee (*Pan troglodytes*), orangutan (*Pongo pygmaeus* and *Pongo abelii*) and olive baboon (*Papio anubis*) faces. Stimuli were obtained from photographs taken of chimpanzees at Kumamoto Sanctuary, Kyoto University, or from personal collections. All faces were presented in greyscale and the average luminance of each face was scaled to the average luminance of all faces in each experiment. This was to control for differences in colour hue, luminance and low-level features which may inadvertently bias attention. Chimpanzee threatening faces were categorised into ‘scream’ (higher threat intensity) and ‘bared teeth’ (lower threat intensity) expressions. A chimpanzee scream expression was defined as: “a raised upperlip with lip corners pulled back exposing the upper teeth, lower lip depressed also exposing the lower teeth, and mouth stretched wide open with lips parted” which is thought to reflect “general agonism” [45] (p. 176). A chimpanzee bared-teeth expression was defined as: “an open mouth with lips parted, a raised upper lip, and retracted lip corners functioning to expose the teeth” which occurs “in response to aggression” [45] (p. 175) and likely reflects fear. The scream and bared-teeth facial expression stimulus set was reviewed by a certified Chimp Facial Action Coding System (ChimpFACS) coder (http://www.chimpfacs.com/) who found the expressions consistent with our categorisation. Colour stimuli consisted of two shades of red; ‘light red’ and ‘dark red’. Object stimuli consisted of two chairs against a nondescript background in colour. Control stimuli consisted of scrambled images. Scrambled chair images were composed by randomly shuffling each pixel of the original images to a new position (box scrambling). This ensured content-related information was removed while average brightness levels were maintained. Scrambled face images were composed by calculating the average power spectrum of the original images to generate phase randomised images (phase scrambling). This preserved the same spatial frequency spectrum as the original images [46]. Stimuli pairs were presented at a distance of 114 mm (432 pixels) between their center points The inside edge of each stimulus was presented at 4° from central fixation so that each stimulus was presented unilaterally to the left and right visual fields [47, 48].

### Procedure

**Fig 1:**
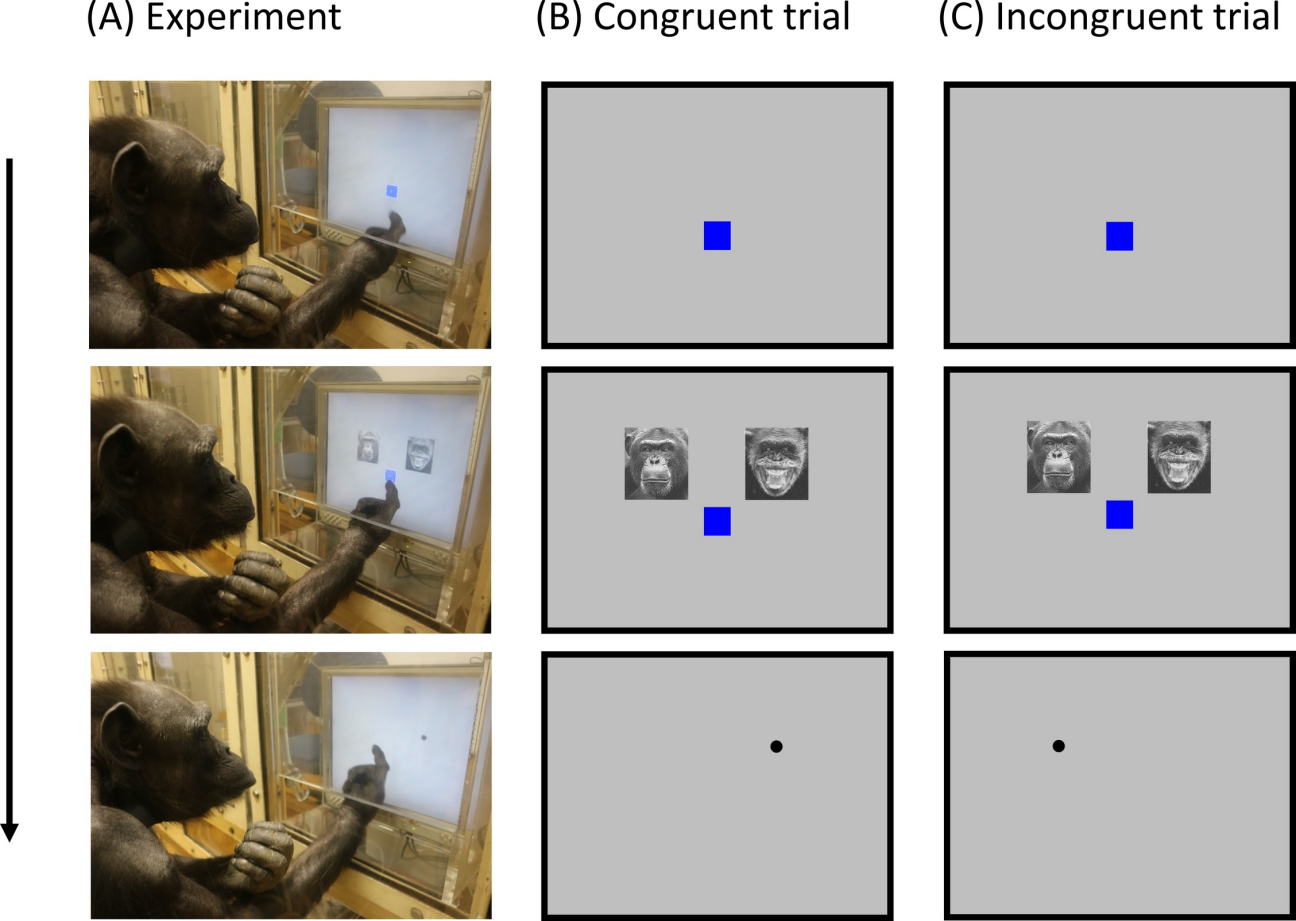
Schematic diagram of the dot-probe task. (A) Experiment: Chimpanzee ‘Ai’ participating in the threatening face experiment. (B) Congruent trial: dot replaces threatening face. (C) Incongruent trial: dot replaces neutral face. Response times (ms) to touch the dot were recorded.

The chimpanzees participated in a series of touchscreen dot-probe tasks (Fig 1). All chimpanzees participated in all experiments which took place every weekday (one session per day). Experiments ran consecutively. To begin each trial the chimpanzees touched a fixation cue (blue square) just below the center of the screen. This was followed by simultaneous presentation of two stimuli directly above the fixation cue. The stimuli and fixation cue then disappeared together and a black dot (probe) appeared immediately in the spatial location of one of the stimuli. The Stimulus Onset Asynchrony (SOA) was 150 ms [29]. When the dot was touched a chime was played and a food reward given. If the dot was not touched it remained on the screen. Response times (ms) to touch the dot were recorded by a PC. The inter-trial interval was 2 s. For each stimuli pair, the dot replaced the stimulus predicted to facilitate faster response times (congruent trials) or the stimulus predicted to lead to slower response times (incongruent trials). In each session half the trials were congruent and half were incongruent. Stimuli presentation (left or right) and congruency (congruent or incongruent dot presentation) was randomised across trials.

### Preliminary experiments

Prior to conducting the main experiment to examine vigilance towards threatening faces, we conducted three preliminary experiments. The aim of these experiments was (a) to examine the sensitivity of the dot-probe task to measure attentional biases towards stimuli of increasing visual, social and emotional complexity and (b) to serve as control experiments to determine that attentional biases measured towards threatening faces are threat-specific and not also found towards non-social or non-threatening stimuli. In Experiment 1 (Colour) we presented two shades of red (two images; dark red and light red) paired with each other or unpaired with any stimuli. Red was chosen because evidence in humans demonstrates that red captures attention and facilitates faster motor responses than non-red cues in the dot-probe task; an effect which is enhanced further for images with an emotional context [49]. We predicted faster response times towards dark red than light red, as there was greater contrast between dark red against the grey background screen than light red. In Experiment 2 (Object) we presented two chairs (two images) paired with scrambled chairs or unpaired with any stimuli. Chairs were chosen as they are not social, threatening or novel for our chimpanzees. We predicted faster response times towards chairs than scrambled chairs, as scrambled chairs lack content-related information. In Experiment 3 (Primate faces) we presented chimpanzee faces (36 images) paired with orangutan (12 images), baboon (12 images) or scrambled chimpanzee (36 images) faces. Primate faces were chosen as they are social but not overtly threatening. In addition, we wanted to build on Tomonaga and Imura’s [39] dot-probe study in chimpanzees which found face-specific attentional biases, by examining whether the task is sensitive to detecting differences in perceptual similarity and familiarity between faces. Chimpanzees are faster at discriminating perceptually different than perceptually similar primate faces [50] and show better performance for discriminating familiar than unfamiliar primate species faces (e.g. [51, 52]). Therefore, we predicted a significant difference in response times for perceptually different faces (chimpanzee paired with baboon) but not for perceptually similar faces (chimpanzee paired with orangutan), with faster response times towards highly familiar faces (chimpanzee) than unfamiliar faces (orangutan or baboon). For Experiment 1, 36 trials × 6 sessions were completed, for Experiment 2, 36 trials × 6 sessions were completed and for Experiment 3, 36 trials × 12 sessions were completed. Trial order was randomised within and across sessions.

### Main experiment

In Experiment 4 (Threatening faces), chimpanzee bared teeth faces (12 images) and scream faces (12 images) were paired with neutral faces (12 novel chimpanzee face images) or scrambled faces (12 images each). For Experiment 4, 48 trials × 12 sessions were completed. Trial order was randomised within and across sessions.

### Statistical analysis

Responses times less than 150 ms and longer than 5,000 ms were excluded from the analysis, as fast response times may have reflected anticipatory responding and slow response times distraction [53, 54]. In addition, for each chimpanzee, condition and session, data two standard deviations above the mean were excluded, resulting in elimination of a total of 513 trials (4.3% of trials). We used R version 3.4.3 [55] and the ‘lme4’ package to perform a Generalized Linear Mixed Model (GLMM) analysis on the relationship between response times and congruency for each stimuli pair comparison. The ‘glmer’ function was used to extract Z values. As fixed effects, we entered congruency and stimuli pair comparison (with interaction term) into the model. The value of the intercept may differ over chimpanzees and sessions, so random intercepts were included. Visual inspection of residual plots revealed the data were skewed, so a gamma probability distribution with a log link function was selected. We chose the model with the smallest AIC values. SPSS (version 24) was also used to analyse response times with repeated measures analysis of variance (ANOVA). Effect sizes were reported with partial etas squared ($\eta_{P}^{2}$).

## Results

### Preliminary experiments

**Fig 2:**
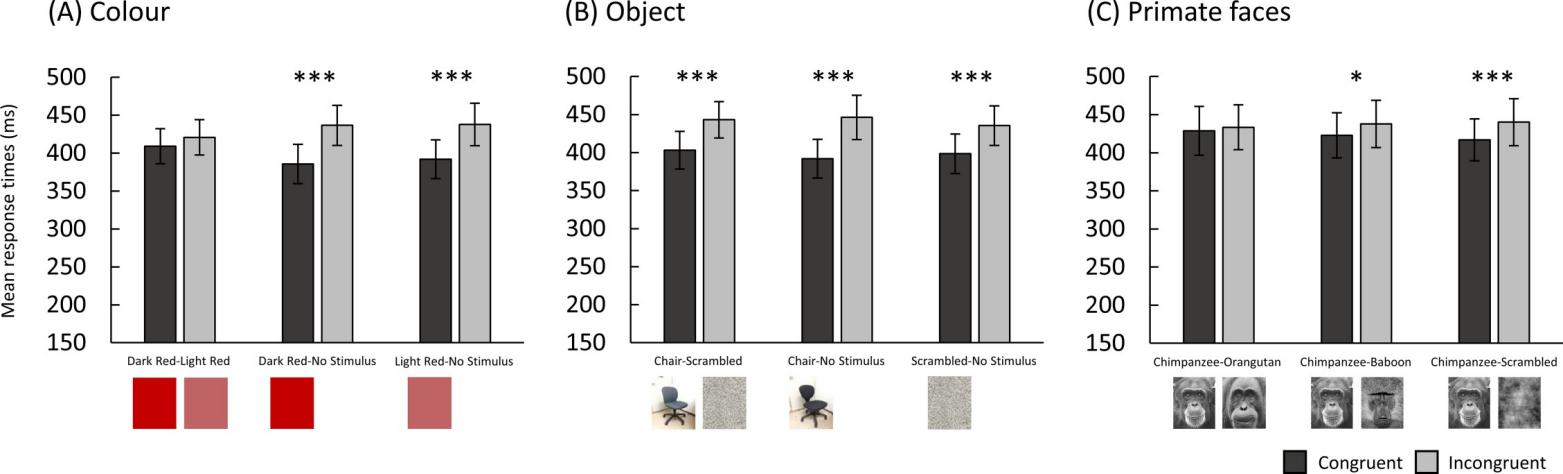
**Mean response times (ms) for congruent and incongruent trials in (A) colour, (B) object, and (C) primate face experiments.** Error bars indicate the standard error of the mean.

Fig 2A. shows the mean response times in Experiment 1 (Colour). Individual chimpanzee data is shown in S1 Dataset. Response times to touch the dot were significantly faster for dark red (congruent, *M* = 386 ms) than no stimulus (incongruent, *M* = 437 ms), (*β* = 0.13, *SE* = 0.02, *Z* = 5.65, *p* < 0.001), and light red (congruent, *M* = 392 ms) than no stimulus (incongruent, *M* = 438 ms), (*β* = 0.12, *SE* = 0.02, *Z* = 5.19, *p* < 0.001). No significant difference in response times was found for dark red (congruent, *M* = 409 ms) versus light red (incongruent, *M* = 421 ms), (*β* = 0.02, *SE* = 0.02, *Z* = 0.98, *p* = 0.329). No other pair comparisons were significant. Fig 2B. shows the mean response times in Experiment 2 (Object). Response times were significantly faster for chairs (congruent, *M* = 403 ms) than scrambled chairs (incongruent, *M* = 443 ms), (*β* = 0.09, *SE* = 0.02, *Z* = 5.54, *p* < 0.001), chairs (congruent, *M* = 392 ms) than no stimulus (incongruent, *M* = 446 ms), (*β* = 0.12, *SE* = 0.02, *Z* = 7.06, *p* < 0.001) and scrambled chairs (congruent, *M* = 398 ms) than no stimulus (incongruent, *M* = 435 ms), (*β* = 0.09, *SE* = 0.02, *Z* = 5.24, *p* < 0.001). No other pair comparisons were significant. Fig 2C. shows the mean response times in Experiment 3 (Primate faces). Response times were significantly faster for chimpanzee faces (congruent, *M* = 423 ms) than baboon faces (incongruent, *M* = 438 ms), (*β* = 0.04, *SE* = 0.01, *Z* = 2.49, *p* = 0.013) and chimpanzee faces (congruent, *M* = 417 ms) than scrambled chimpanzee faces (incongruent, *M* = 440 ms), (*β* = 0.05, *SE* = 0.01, *Z* = 3.79, *p* < 0.001). No significant difference in response times was found for chimpanzee faces (congruent, *M* = 429 ms) versus orangutan faces (incongruent, *M* = 433 ms), (*β* = 0.01, *SE* = 0.01, *Z* = 1.03, *p* = 0.304). No other pair comparisons were significant.

### Main experiment

**Fig 3:**
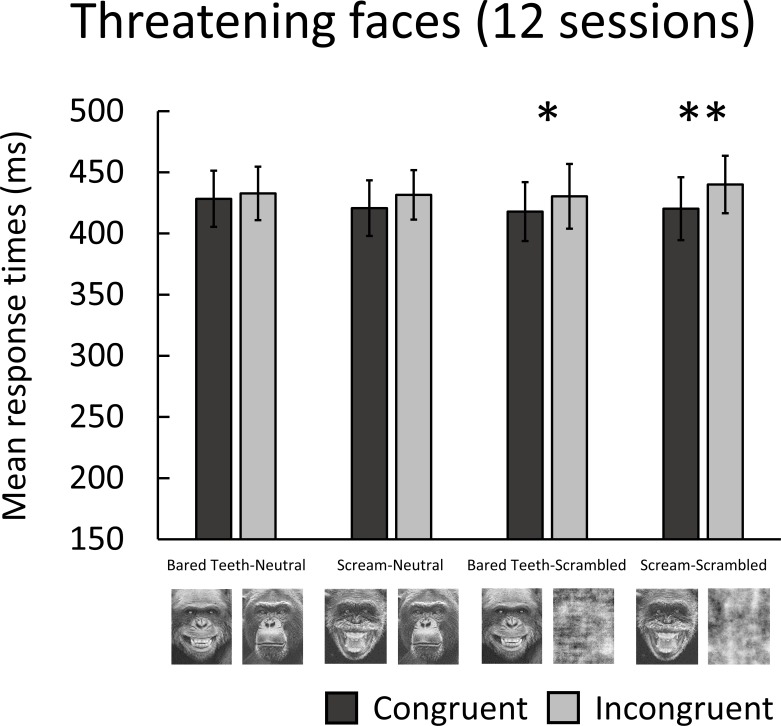
Mean response times (ms) for congruent and incongruent trials in the threatening face experiment. Error bars indicate the standard error of the mean.

Fig 3. shows the mean response times in the threatening faces experiment. Response times were significantly faster for bared teeth faces (congruent, *M* = 418 ms) than scrambled bared teeth faces (incongruent, *M* = 430 ms), (*β* = 0.03, *SE* = 0.01, *Z* = 2.34, *p* = 0.019) and scream faces (congruent, *M* = 420 ms) than scrambled scream faces (incongruent, *M* = 440 ms), (*β* = 0.05, *SE* = 0.01, *Z* = 3.28, *p* = 0.001). No significant difference in response times was found for bared teeth faces (congruent, *M* = 428 ms) versus neutral faces (incongruent, *M* = 433 ms), (*β* = 0.01, *SE* = 0.01, *Z* = 0.61, *p* = 0.544), or scream faces (congruent, *M* = 421 ms) versus neutral faces (incongruent, *M* = 432 ms), (*β* = 0.03, *SE* = 0.01, *Z* = 1.83, *p* = 0.067). No other pair comparisons were significant. No significant difference in response times between bared teeth faces (congruent, *M* = 418 ms) and scream faces (congruent, *M* = 420 ms), (*β* = 0.01, *SE* = 0.01, *Z* = 0.42, *p* = 0.677) paired with scrambled faces was found.

To investigate the possibility that vigilance towards threatening faces may decrease across sessions, we analysed overall response times for each session using Pearson’s Correlation Coefficient. There was no significant relationship between sessions and response times (*M* = 427 ms, *SE* = 23 ms), (*r* = 0.27, *t*_(10)_ = 0.87, *p* = 0.403), indicating no decreasing trend in vigilance across sessions.

**Fig 4:**
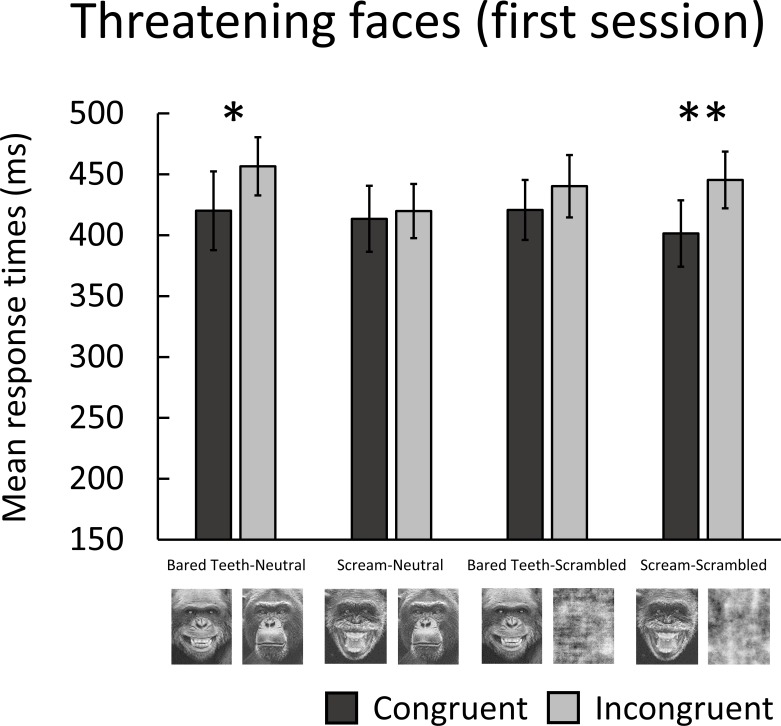
Mean response times (ms) for congruent and incongruent trials in the first session of the threatening face experiment. Error bars indicate the standard error of the mean.

We also explored whether attentional bias effects were more evident at an early stage of the experiment by analysing the mean response times in the first session using GLMM. Fixed effects were congruency and stimuli pair comparisons and the random effect was chimpanzees. Fig 4. shows the mean response times in the first session of the threatening faces experiment. Response times were significantly faster for bared teeth faces (congruent, *M* = 420 ms) than neutral faces (incongruent, *M* = 457 ms), (*β* = 0.09, *SE* = 0.04, *Z* = 2.52, *p* = 0.012), and scream faces (congruent, *M =* 401 ms) than scrambled scream faces (incongruent, *M* = 445 ms), (*β* = 0.10, *SE* = 0.04, *Z* = 2.72, *p* = 0.006). No significant differences in response times were found for scream faces (congruent, *M* = 413 ms) versus neutral faces (incongruent, *M* = 420 ms), (*β* = 0.02, *SE* = 0.04, *Z* = 0.55, *p* = 0.584), or bared teeth faces (congruent, *M* = 421 ms) versus scrambled bared teeth faces (incongruent, *M* = 440 ms), (*β* = 0.04, *SE* = 0.04, *Z* = 1.21, *p* = 0.228). No other pair comparisons were significant. No significant difference in response times between bared teeth faces (congruent, *M* = 420 ms) paired with neutral faces, and scream faces (congruent, *M* = 401 ms) paired with scrambled scream faces (*β* = -0.03, *SE* = 0.04, *Z* = -0.91, *p* = 0.365) was found.

To examine emotional laterality effects, specifically a right brain hemisphere advantage for processing emotional stimuli, reflected in faster response times for threatening faces presented to the left visual field [14, 56–58], we used a 2 × 2 repeated measures ANOVA to compare response times for threatening face (combined bared teeth faces and scream faces) position (left or right visual field) and dot-probe position (left or right) as independent variables. No difference in response times was found between threatening faces presented in the left visual field (*M* = 427 ms, *SE* = 28 ms) and right visual field (*M* = 428 ms, *SE* = 27 ms), (*F*_(1,7)_ = 0.21, *p* = 0.664, $\eta_{P}^{2}$ = 0.028). In addition, no significant difference was found between touching the dot in the left position (*M* = 457 ms, *SE* = 30 ms) and the right position (*M* = 397 ms, *SE* = 25 ms), (*F*_(1,7)_ = 4.45, *p* = 0.073, $\eta_{P}^{2}$ = 0.389), and no interactions were found. Overall, no laterality effects were found.

### Comparison of reaction times across experiments

We further analysed the possibility of increasing or decreasing trends in response times across preliminary and main experiments. A 2 × 3 repeated measures ANOVA was conducted to compare reaction times between experiments with congruency (congruent and incongruent trials) and experiments (1, 2 and 3) as independent variables. To make equivalent comparisons across experiments, only those stimuli paired with scrambled control images were analysed; Experiment 2 (chair–scrambled chair); Experiment 3, (chimpanzee face–scrambled chimpanzee face) and Experiment 4 (combined bared teeth face–scrambled bared teeth face and scream face–scrambled scream face). In addition, only the first six sessions in each experiment were analysed. A significant main effect of congruency was found, with faster response times for congruent trials (*M* = 410 ms, *SE* = 26 ms) than incongruent trials (*M* = 439 ms, *SE* = 27 ms) across experiments, (*F*_(1,7)_ = 13.72, *p* = 0.008, $\eta_{P}^{2}$ = 0.662). However, no significant difference was found in response times between experiments, (*F*_(2, 14)_ = 0.29, *p* = 0.756, $\eta_{P}^{2}$ = 0.039), and no interactions were found. Our chimpanzees showed no systematic trends in response times across experiments.

## Discussion

This study was the first to investigate attentional bias specifically towards threatening faces in chimpanzees using the dot-probe task. In the preliminary experiments, significant biases towards chairs and neutral chimpanzee faces paired with scrambled faces were found. In addition, a significant bias towards highly familiar chimpanzee faces paired with unfamiliar baboon faces was found, but not when chimpanzee faces were paired with unfamiliar orangutan faces. This result is likely explained by the closer perceptual similarity between chimpanzee and orangutan faces than chimpanzee and baboon faces. Wilson and Tomonaga [50] found faster responses for discriminating perceptually different chimpanzee and human faces, or baboon and capuchin monkey faces, than perceptually similar gorilla and orangutan faces. The present study suggests detection of perceptually different faces is possible, even at very short presentation times. In the same chimpanzee group, Tomonaga and Imura [39] found attentional biases towards chimpanzee or human faces paired with random objects, but not towards bananas paired with objects, indicating a face-specific bias. Furthermore, Tomonaga and Imura [59] found chimpanzees rapidly detected faces amongst non-face distractors in a visual search task. Together, these results suggest the dot-probe task is sensitive to detecting a general bias towards faces, as well as larger perceptual differences between faces.

In the main experiment, significant biases towards chimpanzee threatening faces paired with scrambled faces were found. However, there were no significant biases for threatening faces paired with neutral faces, and response times towards threatening faces with higher threat intensity (scream) and lower threat intensity (bared teeth) did not differ. In rhesus monkeys, Parr et al. [38] found a significant bias towards threatening faces versus scrambled images at an SOA of 500 ms. However, these biases may not have been observed if the threatening faces were paired with neutral faces, and so it is unclear whether they are threat-specific. Interestingly, when we analysed the data for the first session, chimpanzees showed an attentional bias towards bared teeth faces (but not scream faces) versus neutral faces. However, this bias disappeared when response times for all sessions were analysed. Similarly, King et al. [37] found a significant bias towards threatening faces versus neutral faces at an SOA of 1000 ms in rhesus monkeys which disappeared over time. It is possible that a threat-specific attentional bias occurred early in our experiment and that repeated exposure to the stimuli weakened this bias, although our shorter SOA of 150 ms may have limited exposure to some extent. Alternatively, this bias could also be explained by individual variation in response times between chimpanzees in the first session. Indeed, biases appear to become more reliable when the dot-probe task is repeated daily over a number of weeks [24]. Overall, in combination with the biases found towards chairs and neutral chimpanzee faces paired with scrambled faces in the preliminary experiments, these results suggest faster response times towards threatening faces reflect a general bias towards faces, rather than threatening faces specifically.

Although we found no convincing evidence for threat-specific attentional biases, it is premature to conclude they do not exist. Several methodological issues may account for our results. One issue may be that the stimuli threat intensity was too weak to facilitate vigilance. In humans, Wilson and MacLeod [32] found morphing angry facial expressions to increase their threat intensity enhanced vigilance. This would be an interesting manipulation to explore in future chimpanzee studies. Another possibility is the stimuli lacked threat intensity as they were presented in greyscale rather than colour. Whilst some evidence suggests chimpanzees can recognise conspecific emotions from greyscale images (e.g., [60]), other evidence suggests effects are only obtained in colour (e.g., [61]). Therefore, in future dot-probe studies it would be useful to test threatening stimuli in colour. In addition, although we were unable to verify the sex of our stimuli, presenting male chimpanzee faces may be more threatening than female faces, as male chimpanzees are more aggressive [62, 63]. Finally, testing chimpanzees with limited exposure to facial stimuli may help to maintain their emotional salience. Our chimpanzees have extensive experience participating in cognitive tasks using facial stimuli, so may be habituated to faces in general.

Another issue is the influence of emotional state on the task. Emotional state has been shown to influence attention in non-human primates [64–66]. Rhesus macaques in a stressful state showed greater avoidance and slower responses to threatening conspecific faces than when in a neutral state [64, 65]. However, these studies presented stimuli for 10–60 seconds and were not restricted to measuring initial threat detection at very short presentation times, as in the present study. In human dot-probe studies, failure to observe vigilance towards threatening faces is often attributed to low trait anxiety or failure to induce a high state of anxiety experimentally [21–23]. Behaviourally, our chimpanzees appeared to be in a relaxed state during the tasks, so anxiety levels may have been too low to facilitate threat-specific biases.

Conversely, attentional training has been shown to influence emotional state in humans (for a review see [67]). In a modified version of the dot-probe task, the dot location is systematically manipulated to increase the proportion of dots appearing in the location of the threatening stimuli or the neutral stimuli. Repeated training leads to an implicitly learned bias towards or away from threat and subsequently an increase or decrease in anxiety respectively (e.g., [68, 69]). In chimpanzees, increasing the predictability of visual social precues (gaze) and non-social cues leads to stronger cueing effects [70, 71]. Therefore, it may be possible to induce a learned bias towards threating faces and subsequently an anxious state in chimpanzees using a modified dot-probe task.

A third issue is the stimuli presentation duration may not have been optimal for facilitating vigilance towards threatening faces. Although we presented stimuli at an SOA of 150 ms based on a review of the human literature (e.g., [18, 26, 27, 29]) this duration may not have been optimal for chimpanzees. Vigilance towards threatening faces has been found at SOAs as short as 17 ms (i.e. subliminal presentation) in humans [72]. However, in chimpanzees Kret et al. [41] failed to find vigilance towards threatening whole-body images presented at 33 ms (also subliminal presentation) and 300 ms. Together, these results suggest the optimal SOA for facilitating vigilance may be very precise and so it would be useful to test additional SOAs in the future.

Finally, manual response dot-probe tasks may not be sensitive enough to detect biases towards threatening faces in chimpanzees. In non-human primates, touchscreen response times are used as an indirect measure of attention. This method assumes gaze location directly corresponds to motor responses. However, more direct measures such as eye tracking and event-related potentials may reveal biases otherwise masked by hand movement and are generally considered more reliable [73–75]. In an example of eye tracking use in non-human primates Pine et al. [76] found monkeys looked significantly longer at threatening than neutral human faces. Bethell et al. [64] observed rhesus macaques were faster to direct initial gaze towards threatening than neutral conspecific faces from video recordings. For further study in chimpanzees, it would be useful to examine to what extent initial gaze location corresponds to manual responses in the dot-probe task using eye tracking.

In conclusion, this study found the touchscreen dot-probe task can measure attentional biases not only towards faces, but perceptually different faces in chimpanzees. However, no evidence for attentional biases specific to threatening faces was found. More research investigating stimuli threat intensity, emotional state, stimuli presentation duration and direct measures of attention is needed to fully explore the potential of the dot-probe task to assess emotional attention in non-human primates.

## Supporting information

S1 Dataset Individual chimpanzee data. (XLSX)
